# A Comparison of the Alvarado Score and the Raja Isteri Pengiran Anak Saleha Appendicitis (RIPASA) Score in the Diagnosis of Acute Appendicitis: A Prospective Cohort Study

**DOI:** 10.7759/cureus.68041

**Published:** 2024-08-28

**Authors:** Kamalesh Inteti, Mohammed Rafi Shaik, Praveena Ganapa, Preethi Shalini Gandi

**Affiliations:** 1 General Surgery, NRI General & Superspeciality Hospital, Guntur, IND; 2 Community Medicine, Kurnool Medical College, Kurnool, IND; 3 Microbiology, NRI General & Superspeciality Hospital, Guntur, IND

**Keywords:** appendicitis diagnosis, : emergency general surgery, modified alvarado vs ripasa, ripasa score, modified alvarado score, diagnosis of acute appendicitis

## Abstract

Background

Diagnosing acute appendicitis remains a problem, especially in teenagers with right lower quadrant pain. Imaging studies aid in accurate diagnosis but have limitations such as cost and availability. The Modified Alvarado Scoring System (MASS) is simple and cost-effective with fewer parameters. The Raja Isteri Pengiran Anak Saleha Appendicitis scoring system (RIPASA), designed for Asian populations, includes more parameters. This study compares the effectiveness of RIPASA and Alvarado scores in diagnosing acute appendicitis in a specific clinical setting.

Objectives

To compare the scoring systems of RIPASA and Alvarado in the diagnosis of acute appendicitis at a tertiary care hospital.

Methods

Data has been collected from all patients who attended the NRI general hospital emergency department and outpatient wing with acute appendicitis, admitted as inpatients based on clinical history and relevant investigations. Patients satisfying inclusion and exclusion criteria were selected and the basic investigations were done. Summary statistics were done using mean, standard deviation and proportions. Inferential statistics were done by using an independent t-test, kappa statistic, sensitivity and specificity with a 95% confidence interval (CI). All the measurements are done using the statistical package for the social sciences (SPSS) software version 21.0 (IBM Corp., Armonk, NY, USA) and open epidemiological (OpenEpi) software 3.01. A probability (p) <0.05 is considered as statistically significant.

Results

A total of 110 patients were analyzed for this study with a majority (39%) of them in the 21-30 age group. In our study, females (53%) outnumbered males (47%). Ultrasound findings in our cohort were acute appendicitis (93%), chronic appendicitis (2%) and normal appendix (5%). However, histopathology reported acute appendicitis (75%), chronic appendicitis (9%) and negative/non-specific (15%). The probability of appendicitis as predicted by Alvarado and RIPASA were 40% and 51% respectively. Definitive diagnosis of appendicitis was made in 16.4% with RIPASA whereas only 5.5% with Alvarado. When comparing the Alvarado and RIPASA scores, the sensitivity or true positive rate was higher for RIPASA (73.63%) than for Alvarado (50.55%).

Conclusion

There was a significant difference between the mean scores in Alvarado and RIPASA inpatients with scores suggestive of appendicitis and no appendicitis. Diagnostic accuracy was higher in RIPASA scoring compared to ALVARDO scoring. There was a significant statistical difference between the two scoring systems. When it comes to diagnosing in low-resource countries the study recommends a combination of Alvarado and RIPASA scoring systems.

## Introduction

The lifetime prevalence of acute appendicitis is approximately one in seven worldwide [1]. It is associated with high morbidity and, occasionally, increased morbidity due to the failure to make an early diagnosis. It is estimated that approximately 6% of the population will suffer from acute appendicitis during their lifetime; therefore, significant effort has been directed toward early diagnosis and intervention [2,3]. In Asian and African countries, the incidence of acute appendicitis is likely lower due to dietary habits prevalent in these regions. Dietary fibre is thought to decrease the viscosity of faeces, reduce bowel transit time, and prevent the formation of fecaliths, which can obstruct the appendiceal lumen [4]. The incidence of appendicitis gradually rises from birth, peaks in the late teenage years, and gradually declines in the geriatric years. It is most prevalent in the 10 to 19-year-old age group [5]. Recently, the number of cases in patients aged 30-69 has increased to 6.3% [6].

Acute appendicitis is the most common surgical abdominal emergency, occurring in 10% of the general population; nevertheless, it remains a diagnostic challenge [7,8]. Several clinical indices and laboratory diagnostic tests can lead to an accurate diagnosis [9]. Traditionally, the diagnosis of appendicitis was based solely on clinical symptoms and signs and later included laboratory results indicating inflammation. This practice led to false-positive cases (negative appendectomy) rates ranging from 15-30% [10-12]. The symptoms of appendicitis overlap with several other conditions, making diagnosis particularly challenging at an early stage [13]. Clinical prediction rules quantify the diagnosis of a target disorder based on key symptoms, signs, and available diagnostic tests, providing independent diagnostic or prognostic value [14]. A negative appendectomy, defined as the removal of an appendix without acute inflammation, is considered acceptable at a rate of 10-20% and can be reduced with the use of imaging studies [15].

Removing a normal appendix is an economic burden on both patients and healthcare resources. Misdiagnosis and delays in surgery can lead to complications like perforation and, ultimately, peritonitis [16]. Acute appendicitis can only be confirmed through histological examination of the resected appendix. Ultrasound, computed tomography (CT), and magnetic resonance imaging (MRI) can markedly assist in diagnosis in many cases [17]; however, ultrasound is limited in obese patients, those with severe abdominal pain, and in cases of retrocecal and perforated appendices, as well as in very thin patients as it fails to visualize the peri-appendiceal inflammation [18]. CT and MRI are relatively expensive and are not readily available in all centres. CT is further discouraged as a choice, if possible, in younger patients due to radiation exposure concerns. Additionally, in the presence of clinical suspicion, a negative radiological examination cannot rule out acute appendicitis [19].

Many scoring systems for the diagnosis of acute appendicitis have been tested, but most are complex and not feasible in an emergency setting. These scores utilize clinical history, physical examination, and laboratory findings. The Modified Alvarado Scoring System (MASS) has been shown in recent studies to be an easy, simple, and cost-effective tool for supporting the diagnosis of acute appendicitis, particularly for junior surgeons [3,19,20]. However, differences in diagnostic accuracy have been observed when the scores are applied to various populations and clinical settings. The Raja Isteri Pengiran Anak Saleha Appendicitis (RIPASA) scoring system is a new diagnostic tool developed for the diagnosis of acute appendicitis and has demonstrated significantly higher sensitivity, specificity, and diagnostic accuracy, especially when applied to Asian populations [21].

The RIPASA scoring system includes more parameters than the Alvarado system, which does not account for factors such as age, gender, and duration of symptoms before presentation. These parameters affect the sensitivity and specificity of the Alvarado scoring system in diagnosing acute appendicitis. The RIPASA system includes 15 diagnostic parameters and has been studied in both Eastern and Western populations [22].

Few studies have compared the RIPASA and Alvarado scoring systems in diagnosing acute appendicitis. In this study, we conducted a comparative prospective study between the Alvarado and RIPASA scores to assess their effectiveness in diagnosing acute appendicitis in our setting.

## Materials and methods

This study took place at NRI (Non-Resident Indian) General and Super Speciality Hospital, a tertiary care centre serving the people of Mangalagiri, Andhra Pradesh, India. Patients attending NRI General Hospital's emergency medical department or outpatient department (OPD) and admitted as inpatients with acute appendicitis during the study period of 18 months, from October 2021 to March 2023, were the source of data collection. Children below 15 years of age, pregnant women, patients with a right iliac fossa mass, patients with a previous history of urolithiasis, and patients with pelvic inflammatory disease were excluded from this study. This is a prospective cohort study comparing two scoring systems against histology-confirmed acute appendicitis.

Patients within the age group of 15-60 years were scored according to the Alvarado and RIPASA scoring systems. The Raja Isteri Pengiran Anak Saleha Appendicitis (RIPASA) is a new diagnostic scoring system developed for the diagnosis of acute appendicitis. The Alvarado score consists of eight parameters, whereas the RIPASA score includes 18 parameters. The scores range from 0.5 to 2 for RIPASA and 1 to 2 for the Alvarado score for each parameter. The scoring charts were filled out by the attending surgeon at the time of presentation. A score of 7 was considered to indicate a high probability of acute appendicitis in the Alvarado scoring system, while a score of 7.5 indicated a high probability in the RIPASA scoring system. The decision to perform an appendectomy was based solely on the surgeon's clinical judgment after considering all the findings from clinical, laboratory, and radiological investigations. The parameters for Alvarado's score and RIPASA score are depicted in Table 1 and Table 2, respectively.

**Table 1:** ALVARADO Score

Parameter	Score
Migratory pain to RIF (Right iliac fossa)	1
Anorexia	1
Nausea & Vomiting	1
RIF tenderness	2
Rebound tenderness	1
Fever	1
Raised WBC (White blood cells)	2
Shift of WBC to left	1
Total	10

**Table 2:** RIPASA score

Study parameter	Score
Demographic information	
Female	0.5
Male	1
Age <40	1
Age >40	0.5
Foreign national	1
Symptoms	
Right iliac fossa (RIF) pain	0.5
Migratory pain to RIF	0.5
Anorexia	1
Nausea & Vomiting	1
Duration of symptoms <48 hrs	1
Duration of symptoms >48 hrs	0.5
Signs	
RIF tenderness	1
Guarding	2
Rebound tenderness	1
Rovsing sign	2
Temperature between 37 and 39	2
Investigations	
Elevated white blood cell count	1
Negative urine analysis (absence of blood, WBCs, bacteria)	1
Maximum Score	17.5

The RIPASA and Alvarado scores were used solely for the study's purpose. Patients were monitored following admission, surgery, and until discharge from the hospital. Daily follow-up included monitoring of vitals three times a day and a systemic examination once a day. Scores were tabulated and compared using the chi-square test. The demographics of all patients and the distribution of patients by individual scoring systems, RIPASA and Alvarado, were tabulated.

Summary statistics were calculated using mean, standard deviation, and proportions. Inferential statistics were performed using the independent t-test, kappa statistic, sensitivity and specificity with 95% confidence intervals (CI), and receiver operating characteristic (ROC) curve analysis with the area under the curve. All measurements were conducted using a statistical package for the social sciences (SPSS) software version 21.0 (IBM Corp., Armonk, NY, USA) and open epidemiological (OpenEpi) software 3.01, with a probability value (p-value) < 0.05 considered statistically significant. Institutional ethical clearance (IEC) was obtained before the commencement of this study. Informed consent was obtained from all participants. No ethical concerns were raised in this study, and approval was obtained from the local clinical governance team. The data collected did not include any identifiers, and confidentiality was ensured. The authors have no conflicts of interest.

## Results

A total of 110 patients attending NRI General Hospital's emergency medical department, outpatient department (OPD), or admitted as inpatients for acute appendicitis during the study period of 18 months were included. All patients were between 15-60 years of age and met the inclusion criteria.

Among the 110 patients, the majority were between the ages of 21-30 years, accounting for 43 patients (39.1% of the total sample). Only 14 patients were older than 50 years and under 20 years, each representing 12.7% of the total sample. The remaining age groups were distributed as follows: under 20 years - 14 (12.7%), 31-40 years - 23 (20.9%), and 41-50 years - 16 (14.5%). Table 3 describes the age distribution among the patients.

**Table 3:** Age group distribution among the patients

Age (in years)	Frequency	Percentage (%)
< 20	14	12.7
21-30	43	39.1
31-40	23	20.9
41-50	16	14.5
>50	14	12.7
Total	110	100

Table 4 shows the gender distribution of the study population. The majority were females (52.7%) compared to males (47.3%).

**Table 4:** Gender distribution among patients

Age (in years)	Frequency	Percentage (%)
Male	52	47.3
Female	58	52.7
Total	110	100

Table 5 shows the ultrasonographic findings of the patients. Among the 110 patients, 90% had acute appendicitis, 2.7% had acute suppurative appendicitis, 5.5% had a normal appendix but with other parameters suggestive of acute appendicitis, and 1.8% had chronic appendicitis.

**Table 5:** Ultrasonography findings among patients

Condition	Frequency	Percentage (%)
Acute appendicitis	99	90
Acute suppurative appendicitis	3	2.7
Chronic appendicitis	2	1.8
Normal appendix	6	5.5
Total	110	100

In Table 6, the histopathology reports of the patients subjected to appendectomy were reviewed. The majority, about 50.9%, had acute appendicitis, and about 9% had acute suppurative appendicitis. Other histopathological findings included chronic appendicitis (7.2%) and follicular hyperplasia (9.1%) in a minority of patients.

**Table 6:** Histopathological findings among patients

Histopathological finding	Frequency	Percentage (%)
Acute appendicitis	56	50.9
Acute appendicitis with mesoappendicitis	1	0.9
Acute appendicitis with periadenitis	6	5.5
Acute appendicitis with periappendicitis	5	4.5
Acute or chronic	6	5.5
Acute suppurative	10	9.1
Acute suppurative with appendicitis	4	3.6
Acute suppurative appendicitis with reactive follicular hyperplasia	1	0.9
Chronic appendicitis	8	7.22
Chronic appendicitis with follicular hyperplasia	1	0.9
Follicular hyperplasia	10	9.1
Subacute appendicitis	2	1.8
Total	110	100

The mean age in our study group was 34.1 years with a standard deviation of ±13.69 years. The mean total leukocyte count (TLC) was 10,370.09 cells/cumm with a standard deviation of ±3872.75 cells/cumm. The mean TLC count in patients with appendicitis was 10,624.51 cells/cumm with a standard deviation of ±3936.87, whereas in patients without appendicitis, it was 9151 cells/cumm with a standard deviation of ±3381. There was no significant difference in the total leukocyte count between patients with appendicitis and those without (p=0.104).

Table 7 shows the Alvarado scoring among patients. About 40% had a score suggesting probable appendicitis, and only six subjects (5.5%) had a score above 9, indicative of confirmed cases. About 28.2% and 26.4% of patients had scores in the "not sure" and "compatible" categories, respectively.

**Table 7:** ALVARADO scoring among patients

ALVARADO Score	Frequency	Percentage (%)
Not sure	31	28.2
Compatible	29	26.4
Probable	44	40
Confirmed	6	5.5
Total	110	100

**Figure 1:**
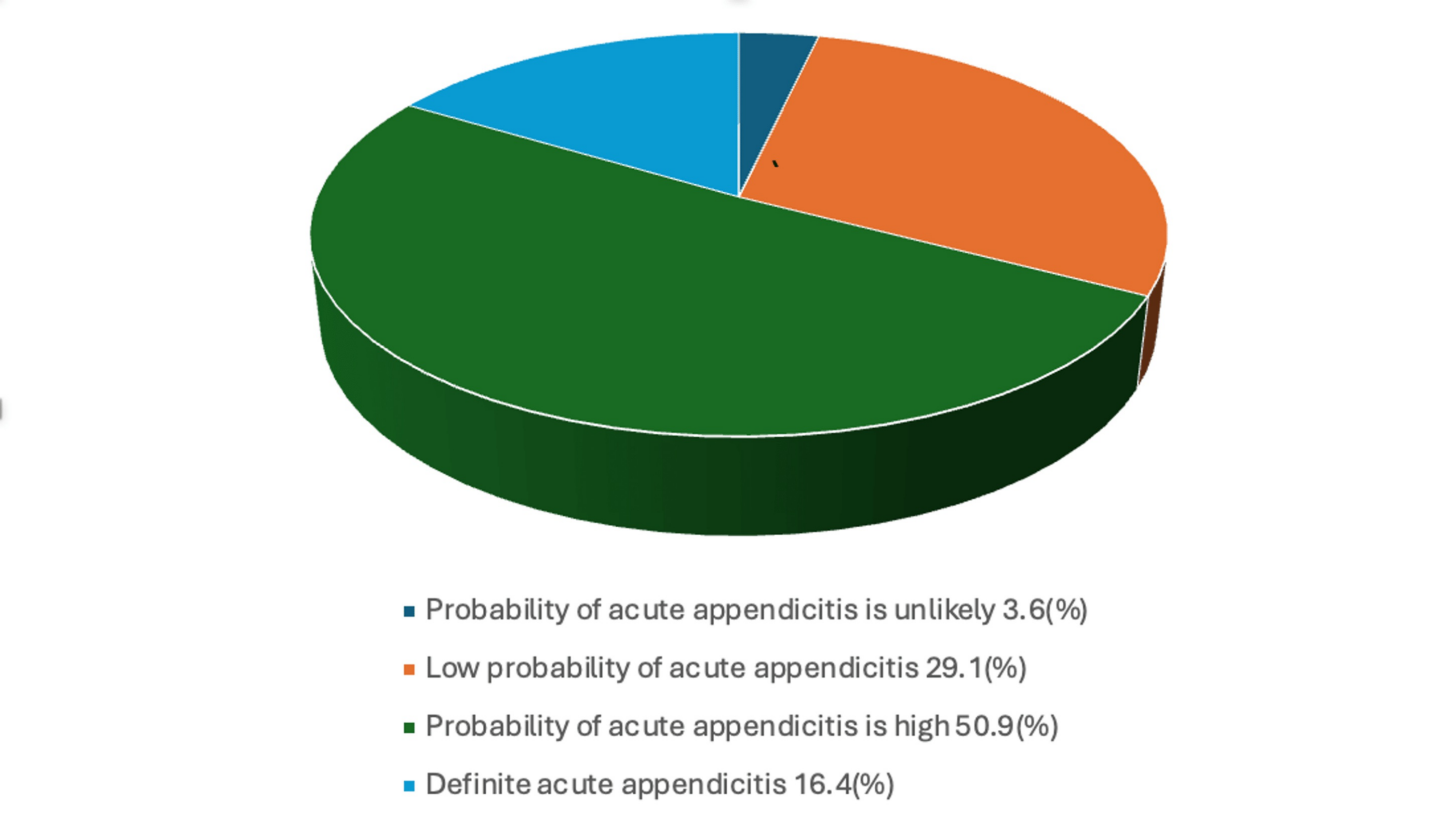
RIPASA scoring among patients

When the RIPASA score was applied to patients with clinically suspected appendicitis, about 50.9% were categorized under a high probability of appendicitis, and 16.4% had scores indicative of confirmed appendicitis. About 29.1% had scores suggesting a low probability of appendicitis, as displayed in Figure 1.

The sensitivity of the Alvarado score was estimated to be 50.55%, with a 95% confidence interval ranging from 40.46% to 60.59%. The specificity, positive predictive value, and negative predictive value are described in the table with a 95% confidence interval. The diagnostic accuracy of the Alvarado scoring was found to be 55.45%, with a 95% confidence interval ranging from 46.14% to 64.4%. When comparing the Alvarado and RIPASA scores, the sensitivity or true positive rate was higher for RIPASA (73.63%) compared to Alvarado (50.55%). However, the true negative rate or specificity was higher for the Alvarado scoring system.

**Figure 2:**
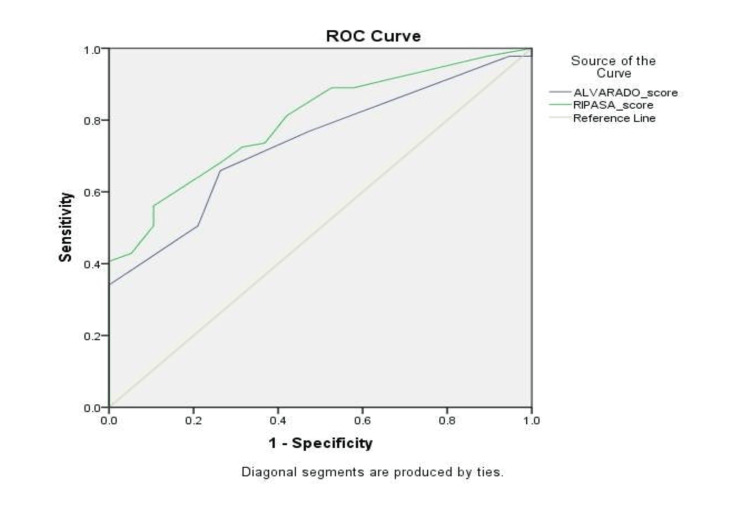
ROC curve receiver operating characteristic curves with the corresponding area under the curve (AUC) for ALVARADO and RIPASA scoring system in predicting acute appendicitis

The positive predictive values were more or less equal for both the Alvarado and RIPASA scoring systems. The negative predictive value was higher for RIPASA compared to Alvarado. A difference of 16.4% was observed in diagnostic accuracy, with RIPASA showing a higher accuracy rate of 71.82% compared to Alvarado's 55.45%. This is displayed under the receiver operating characteristic (ROC) curve in Figure 2.

## Discussion

Age

In a prospective study by Nanjundaiah et al., the mean age of 206 patients admitted with right iliac fossa pain was 27.82 ± 9.262 years. Among them, 52.7% were females and 47.3% were males [23]. In a cross-sectional study by Zulfiqar et al. on 250 patients with acute appendicitis, the mean age was 35.17 ± 9.13, similar to our study. Our study's mean age correlates with the findings of Zulfiqar et al. [24] and Kothari et al. [25] but is higher compared to Nanjundaiah et al. [23]. A cross-sectional study by Kothari et al. on 80 patients reported an age range from 11 to 72 years (mean 32.89 ± 15.87), comparable to our study [25]. In a study by Khan et al., the mean age was 20.2 years [26,27]. The mean age in years for patients with appendicitis in our study was 35.16 years with a standard deviation of 14.32 years, whereas for patients without appendicitis, it was 29.47 years with a standard deviation of 9.02 years. The mean age showed a significant difference between patients with and without appendicitis (p=0.032).

Gender

The study included 52.7% females and 47.3% males. Although females were numerically the majority, this was not statistically significant. A time-bound prospective study by Kapoor et al. [28] on 50 cases of suspected acute appendicitis found a similar distribution, with males comprising 56% and females 44%. Khan et al. [26] reported a male-to-female ratio of 1:1.44, while Talukder et al. [29] found that males were more susceptible than females, with a male-female ratio of 1.38:1.

Ultrasonography

Among the 110 patients in our study, 90% had acute appendicitis, 2.7% had acute suppurative appendicitis, 5.5% had a normal appendix, and 1.8% had chronic appendicitis. In a prospective study by Kapoor et al. [28] on 50 patients with suspected acute appendicitis, ultrasonography showed positive results in 41 cases (82%), with 80% showing a congested and edematous appendix and 2% having a perforated appendix. No abnormalities were detected in 18% of cases, comparable to our findings.

Histopathology

In our study, 82.7% of patients had appendicitis based on histopathological reports, while 17.3% did not. The difference of 7.3% between ultrasonography and histopathology in diagnosing acute appendicitis was not significant. Histopathology revealed that 50.9% of patients had acute appendicitis, 9% had acute suppurative appendicitis, 7.2% had chronic appendicitis, and 9.1% had follicular hyperplasia.

The mean total leukocyte count (TLC) in our study was 10,370.09 cells/cumm with a standard deviation of ±3872.75 cells/cumm. There was no significant difference in TLC between patients with appendicitis (mean 10,624.51 cells/cumm, Standard deviation (SD) ±3936.87) and those without appendicitis (mean 9151 cells/cumm, SD ±3381) (p=0.104). However, the main difference spotted was the use of differential leucocyte count in Alvarado which scores high when compared to RIPASA which included total leucocyte count.

ALVARADO

Patients with an Alvarado score between 6-9 or above 9 were considered to have a high probability of appendicitis. In our study, 45.5% of patients had a high probability of confirmed appendicitis based on Alvarado scores, while 60 patients (54.5%) had lower scores, suggesting clinically suspected appendicitis.

RIPASA

Approximately 67.3% of patients clinically suspected of having appendicitis had RIPASA scores indicating probable or confirmed appendicitis, while 32.7% had scores suggesting low probability. In a study by Nanjundaiah et al. [23], 87.4% had RIPASA scores >7, indicating appendicitis and 12.6% had scores between 5-7.

The mean Alvarado score for patients with appendicitis was 6.45 (SD 1.97), and for those without appendicitis, it was 4.89 (SD 1.28). The mean RIPASA score for patients with appendicitis was 9.43 (SD 2.68) compared to 6.68 (SD 1.73) for those without. Significant differences were found between the mean Alvarado and RIPASA scores for patients with and without appendicitis. The sensitivity of the Alvarado score in our study was estimated at 50.55, with a 95% confidence interval of 40.46-60.59. The diagnostic accuracy was 55.45, with a 95% confidence interval of 46.14-64.4.

In Nanjundaiah et al.'s study [23], an ALVARDO score cutoff of >7 showed sensitivity and specificity of 58.9% and 85.7%, respectively, similar to our findings. The positive predictive value (PPV) and negative predictive value (NPV) for the Alvarado score were 97.3% and 19.1%, respectively, aligning with our results.

For the RIPASA score, our study showed a sensitivity of 73.63, specificity of 63.16, PPV of 90.54, and NPV of 33.33. The diagnostic accuracy of the RIPASA score was 71.82, with a 95% confidence interval of 62.78-79.38. Nanjundaiah et al. [23] reported that with a RIPASA score cutoff of >7.5, sensitivity and specificity were 96.2% and 90.5%, respectively. The PPV and NPV were 98.9% and 73.1%, respectively.

Validity comparison between Alvarado and RIPASA scoring systems: Our study observed a 16.4% difference in diagnostic accuracy between the Alvarado and RIPASA scores, with RIPASA showing higher accuracy (71.82% vs. 55.45%). Nanjundaiah et al. [23] found a 33.93% difference.

Comparison between ALVARDO and RIPASA scoring systems: There was a significant statistical difference between the Alvarado and RIPASA scores in our study (p < 0.0001), with a Kappa value of 0.401. This finding is comparable to the study by Chong et al. [30].

ROC curve: The ROC curve analysis showed that the area under the curve (AUC) was higher for the RIPASA scoring system compared to the Alvarado system. The AUCs for both scoring systems were significant. In Nanjundaiah et al.'s study [23], a significant 13.4% difference in AUC between the two scoring systems indicated that the Alvarado score misdiagnosed 13.4% more patients with acute appendicitis compared to the RIPASA score.

## Conclusions

The inclusion of additional parameters in the RIPASA score provides greater flexibility and adaptability to different geographical regions. Implementing the Alvarado and RIPASA scores serves as an adjunct to clinical examination however neither in isolation nor combination appears to change clinical impression in most patients with suspected appendicitis. They appear to have a role in helping support clinical impressions regarding the likelihood of acute appendicitis. However, this study recommends including additional parameters in RIPASA to improve the accuracy of the scoring system. Future prospective studies are needed to confirm these findings and further validate the use of these scoring systems in different populations and settings.
